# Insights into knowledge, attitudes, and practices of medicinal plant use in the United Arab Emirates: a cross-sectional study

**DOI:** 10.3389/fnut.2026.1755440

**Published:** 2026-03-16

**Authors:** Seham M. Al Raish, Hind N. Alsheriafi, Aysha A. Alkuwaiti, Samir K. Safi, Ali S. Safi

**Affiliations:** 1 Department of Biology, College of Science, United Arab Emirates University, Al Ain, United Arab Emirates; 2 Department of Statistics and Business Analytics, CBE, United Arab Emirates University, Al Ain, United Arab Emirates

**Keywords:** herbal medicine, knowledge-attitude-practice, medicinal plants, sociodemographic factors, traditional medicine, UAE

## Abstract

**Background:**

The use of medicinal plants remains an important component of traditional and complementary medicine in the United Arab Emirates (UAE). However, comprehensive studies evaluating public knowledge, attitudes, and practices (KAP) regarding herbal medicine are limited. This study aims to evaluate KAP toward medicinal plant use in the UAE and examine the influence of sociodemographic factors.

**Methods:**

A cross-sectional online survey was conducted among 418 participants. The reliability and validity of the KAP questionnaire were evaluated using Cronbach’s alpha (*α* = 0.870) and split-half reliability (0.794). Descriptive statistics, correlation analysis, and regression models were employed to analyze the data. The Kruskal–Wallis test was applied to assess differences across sociodemographic groups.

**Results:**

The majority of respondents (75%) had knowledge scores above the scale midpoint (>2.5 on a 4-point Likert scale), with higher scores among males (*p* < 0.001) and middle-aged individuals (25–54 years). Positive correlations were observed between knowledge and attitude (*r* = 0.659, *p* < 0.001), knowledge and practice (*r* = 0.501, *p* < 0.001), and attitude and practice (*r* = 0.691, *p* < 0.001). Regression analysis indicated that knowledge and practice significantly predicted attitude (*R*^2^ = 0.631, *p* < 0.001). Regular herbal medicine users had significantly higher KAP scores (*p* < 0.001), with the strongest effect observed in the practice domain (η^2^ = 0.148).

**Conclusion:**

The UAE population demonstrates generally positive knowledge, attitudes, and practices toward medicinal plants, influenced by age, gender, education, and usage frequency. These findings highlight the importance of targeted public health education to promote the safe and evidence-based use of herbal medicine. Ethical approval was obtained from the United Arab Emirates University (UAEU) Social Sciences Ethics Committee (Research No: ERSC_2025_5931; approved on 01/03/2025).

## Graphical abstract

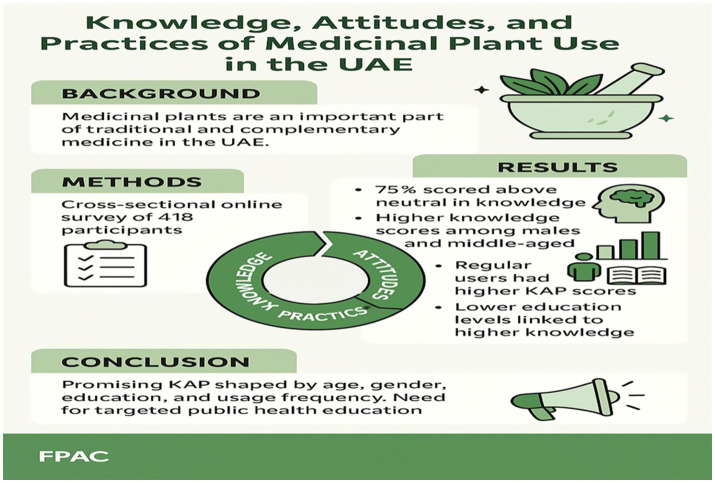

## 1 Background

Medicinal plants (MP) have long played a key role in traditional and complementary healthcare systems worldwide (1–3). Despite advances in pharmaceutical development, plant-based remedies remain widely utilized due to cultural traditions, accessibility, and perceived safety (4–6).

The World Health Organization estimates that a substantial proportion of individuals in developing countries rely on traditional or plant-based medicine for disease prevention and treatment (7–9). Recent experimental and clinical research has further highlighted the therapeutic relevance of medicinal plants, including commonly used species such as *Allium cepa* and *Allium sativum* (10), *Nigella sativa* and *Ziziphus lotus* (11), as well as several medicinal plants native to the UAE that exhibit antidiabetic potential (*Capparis spinosa*, *Citrullus colocynthis*, *Morus alba*, *Rhazya stricta*) (12) and polyphenol-rich plant interventions with metabolic benefits (13). These findings reinforce the importance of understanding how such plants are perceived and used in the community (14, 15).

Although medicinal plants are widely used, public understanding of their safety, efficacy, and potential interactions with conventional medicine varies considerably (16–18). Misconceptions regarding safety and therapeutic scope may contribute to inappropriate self-medication, delayed clinical consultation, and potential drug–herb interactions (19–21). Therefore, assessing knowledge, attitudes, and practices (KAP) regarding medicinal plant use is essential for promoting safe consumption and guiding regulatory and educational strategies (22–24).

In the Middle East, herbal medicine use remains culturally and socially embedded. Studies conducted in Saudi Arabia, Jordan, and Iran have reported high prevalence of herbal medicine use alongside generally positive public attitudes (25–28). However, these studies have also highlighted gaps in safety awareness, inconsistent communication with healthcare providers, and variability in knowledge levels across demographic groups. These findings underscore the need for structured evaluation of medicinal plant–specific KAP in different national contexts, particularly in rapidly developing healthcare systems.

Within the Gulf region, research has largely focused on complementary and alternative medicine (CAM) broadly rather than medicinal plants specifically (29–32). In the United Arab Emirates (UAE), Aljawarneh et al. reported generally favorable knowledge and attitudes toward CAM but identified gaps related to safety and risk perception (33). However, medicinal plant–specific KAP has not been independently assessed in the UAE. Given the UAE’s culturally diverse population, increasing accessibility of herbal products, and evolving regulatory landscape, a focused evaluation of medicinal plant–related KAP is warranted (30, 34, 35).

Understanding how knowledge relates to attitudes and practices, and how sociodemographic factors influence these domains, is essential for informing public health education, regulatory oversight, and culturally appropriate policy development (36–38).

Therefore, this study aims to:

1. Evaluate knowledge, attitudes, and practices regarding medicinal plant use among UAE residents.
2. Examine the interrelationship between knowledge, attitudes, and practices.
3. Investigate the influence of sociodemographic factors on KAP scores.

We hypothesize that UAE residents will demonstrate moderate to high levels of knowledge regarding medicinal plants; that knowledge will be positively associated with attitudes and practices; and that sociodemographic variables will significantly influence KAP outcomes.

## 2 Methods

### 2.1 Study design and data collection

This study employed a descriptive, analytical, cross-sectional design using an online self-administered questionnaire. Ethical approval was obtained from the United Arab Emirates University (UAEU) Social Sciences Ethics Committee (Research No: ERSC_2025_5931; approved on 01/03/2025). All participants provided informed consent prior to participation and were informed of their right to withdraw at any time.

Participants were recruited through online distribution using a random dissemination approach. Inclusion criteria were residence in the UAE and age between 18 and 64 years. Individuals under 18 years and non-residents were excluded (Tables 1–3).

**Table 1:** Knowledge questions.

Code	Item statement	References
KQ1	Herbal medicines are made from plant sources	(39, 40)
KQ2	Herbal medicines can prevent all diseases	(40)
KQ3	Herbal medicines can cure all diseases	(40)
KQ4	Herbal medicine is always safe	(39, 40)
KQ5	Overuse of herbal medicine can cause adverse effects	(40)
KQ6	Herbal medicines can be taken with conventional or allopathic medicines	(40)
KQ7	Herbal medicines do not expire	(40)

KQ, knowledge question.

**Table 2:** Attitude questions in the questionnaire.

Code	Item statement	References
AQ1	Herbal medicines are safe because they are made from natural ingredients	(39, 40)
AQ2	Herbal medicines are better than conventional medicines	(40)
AQ3	Many health claims made by herbal medicine manufacturers are unproven	(40)
AQ4	I prefer herbal medicines because they are cheap and easily available	(40)
AQ5	It is important to consult a doctor or pharmacist before using herbal medicines	(40)
AQ6	I trust published information on traditional recipes in commercial channels	(40)
AQ7	The effect of herbal medicine is usually due to placebo	(41)
AQ8	Herbal medicine should be further researched and evaluated by universities	(41)
AQ9	If herbal medicine has equal effect as a chemical drug, I would choose herbal medicine	(41)
AQ10	Herbal medicine education should be included in continuing medical education programs	(41)

AQ, Attitude question.

**Table 3:** Practice questions in the questionnaire.

Code	Item statement	Response format	Reference
PQ1	When I get sick, I first take herbal medicines	4-point Likert scale	(39, 40)
PQ2	I take herbal medicines for acute conditions (e.g., severe pain)	4-point Likert scale	(39, 40)
PQ3	I always check the expiry date before taking herbal medicines	4-point Likert scale	(39, 40)
PQ4	I advise others to use herbal medicines when they have health problems	4-point Likert scale	(39, 40)
PQ5	Source of information about traditional recipes	Multiple choice	(42)
PQ6	Type of traditional recipes used	Multiple choice	(42)

PQ, Practice question.

### 2.2 Sample size determination

The required sample size was calculated using G*Power version 3.1.9.6. Assuming a medium effect size (f^2^ = 0.15), an alpha level of 0.05, statistical power (1 − *β*) of 0.80, and two predictors in a multiple regression model, the minimum required sample size was 107 participants. To enhance representativeness relative to the UAE population and account for potential non-response, the final sample was expanded to 418 participants.

### 2.3 Questionnaire structure and scoring

The questionnaire was developed based on previously validated KAP instruments (39–42) and consisted of two sections. The first section included six sociodemographic questions. The second section comprised 23 items assessing knowledge (7), attitudes (10), and practices (6) related to medicinal plant use.

All knowledge and attitude items and four practice items (PQ1–PQ4) were measured using a four-point Likert scale (1 = strongly agree to 4 = strongly disagree). Two practice items (PQ5 and PQ6) were categorical multiple-choice questions and were analyzed descriptively.

Domain scores were computed as the mean of their respective Likert-scale items. Negatively worded statements (e.g., “Herbal medicines can prevent all diseases,” “Herbal medicines can cure all diseases,” and “Herbal medicine is always safe”) were reverse-coded prior to analysis to ensure that higher scores consistently reflected more accurate knowledge or more positive attitudes and practices.

Internal consistency analysis included only the 21 Likert-scale items (7 knowledge, 10 attitude, and 4 practice items).

## 3 Statistical analysis

### 3.1 Reliability analysis of the questionnaire

The reliability analysis was performed using two methods: Cronbach’s alpha and the split-half method. The Cronbach’s alpha for the knowledge domain was 0.502, indicating unacceptable reliability. However, the analysis suggests that removing paragraph 4, “Herbal medicines are preferred because of fewer side effects,” would increase the Cronbach’s alpha for the knowledge domain to 0.674. Based on this result, the paragraph was excluded from the final questionnaire and was not included in any further statistical analyses.

The Practice domain demonstrated modest internal consistency (Cronbach’s *α* = 0.592; Omega ML = 0.595; Omega HA = 0.572), which is below the conventional 0.70 threshold. This may be attributable to the limited number of paragraphs and the inherently heterogeneous nature of behavioral measures, where practices may not be strongly intercorrelated. Although the overall scale showed strong reliability (*α* = 0.870), the Practice subscale’s lower reliability should be interpreted with caution.

Table 4 shows the updated reliability analysis results for the knowledge, attitude, and practice (KAP) domains, as well as for all items within these domains. The revised results show Cronbach’s alpha, McDonald’s Omega (ML), and McDonald’s Omega (HA) values of 0.870, 0.874, and 0.867, respectively, for the overall 21 paragraphs of the questionnaire.

**Table 4:** Reliability analysis of KAP and the overall 21 paragraphs of the questionnaire.

Domain	Split-half	Cronbach’s alpha	Omega ML	Omega HA
Knowledge	0.597	0.674	0.710	0.687
Attitude	0.747	0.784	0.786	0.775
Practice	0.604	0.592	0.595	0.572
Overall	**0.794**	**0.870**	**0.874**	**0.867**

*Overall represents the reliability estimates computed for all domains combined (Knowledge, Attitude, and Practice).

The split-half reliability coefficient (0.794) was calculated using the odd–even splitting method, whereby items were divided into two halves based on their sequence order (odd-numbered items vs. even-numbered items). The correlation between the two halves was then computed and adjusted using the Spearman–Brown prophecy formula to obtain the final split-half reliability estimate. These values significantly exceed the threshold of 0.70 for acceptable reliability (47). Thus, these results show that the items in the scale are highly intercorrelated and likely measure the same underlying construct effectively. Detailed results for each KAP domain are provided in Table 4.

### 3.2 Sociodemographic variables

Table 5 presents the sociodemographic characteristics of study participants (*N* = 418). The survey’s age distribution indicates that most respondents (58.4%) were in the 18–24 age range. Participants aged 55–64 (1.2%) had a smaller proportion. This pattern probably indicates that online platforms are more accessible and that younger people are more willing to participate in surveys. The gender distribution of the participants was nearly equal, with 50.2% identifying as female and 49.8% as male.

**Table 5:** Sociodemographic characteristics of the questionnaire.

Variable	Categories	Frequency	Percent
Age	18–24 years old.	244	58.37
	25–34 years old	89	21.29
	35–44 years old.	58	13.88
	45–54 years old.	22	5.26
	55–64 years old.	5	1.20
Gender	Male	208	49.76
	Female	210	50.24
Education	Less than high school	6	1.44
	High school degree	132	31.58
	Bachelor’s degree	236	56.46
	Master’s degree	39	9.33
	Ph.D.	5	1.20
Employment	Employed	177	42.34
	Retired	16	3.83
	Student	176	42.11
	Unemployed	49	11.72
Herbal medicine intake	Never	96	22.97
	Rarely	114	27.27
	Often	160	38.28
	Always	48	11.48
Reason of taking herbal medicine	Respiratory illness	103	24.64
	Urinary tract infection	58	13.88
	Abdominal pain	171	40.91
	Dental pain	120	28.71
	Skin problem or wound	156	37.32
	Joint diseases	77	18.42
	Hypertension	57	13.64
	Diabetes	34	8.13
	Metabolic diseases	9	2.15
	Psychology	3	0.72
	Digestive tract	8	1.91
	Vitamins	3	0.72
	Pulmonary disease	2	0.48
	Not Considered	46	11.00
	Total	847*	

*Note that the total of 847 exceeds the sample size of 418 because it is a multiple-response questionnaire.

Regarding education, the majority of respondents (56.5%) had a bachelor’s degree. The percentage claiming a doctorate (1.2%) was the lowest. This distribution suggests that the sample is generally well educated. Regarding employment status, students accounted for the largest share of participants (42.1%), followed by employed people (42.3%). However, only 3.8% of respondents reported being retired. The study’s outreach to a younger population or academic community, where student and early-career participation is more common, is shown in this distribution.

The results show that 11.5% of the respondents claimed they always used herbal medicine, although the majority (38.3%) said they used it often. This could be because herbal medicines are accessible alternatives to conventional medicine, are perceived as safe, or are socially familiar. (40.9%) Of participants said they most commonly used herbal medicine for abdominal pain, and.

(17.0%) of respondents also reported using herbal medicines for a variety of other issues, including, not considered the highest among others (11.0%), then metabolic diseases (2.15%), and the least being pulmonary disease (0.48%).

### 3.3 Descriptive statistics for knowledge, attitude, and practice

The KAP scores were calculated as the mean of the paragraphs within each domain, rather than as a summed total. Specifically, for each participant, the domain score was computed by averaging responses across all items in that domain. Using the mean rather than the sum ensures that the scores remain on the original Likert scale for easy interpretation, and this approach maintains comparability across domains, even when the number of items differs.

The results show that the mean scores for knowledge, attitude, and practice domains equal 2.85, 3.09, and 3.11, respectively.

On a 1–4 Likert scale (midpoint = 2.5), the mean Knowledge score of 2.85 is above the midpoint, indicating an overall tendency toward agreement/adequate knowledge rather than a neutral or low level. Similarly, the Attitude (3.09) and Practice (3.11) means indicate generally positive attitudes and practices.

Regarding variability, the standard deviations (Knowledge = 0.49, Attitude = 0.46, Practice = 0.54) indicate relatively modest dispersion around the means, suggesting moderate clustering of responses rather than substantial heterogeneity. The paragraph *“Herbal medicines do not expire”* (Mean = 2.31) was the only knowledge item with a mean below the scale midpoint, indicating a relatively lower level of correct understanding for that paragraph.

The results show a moderate variation, as indicated by the standard deviations of 0.49, 0.46, and 0.54 for each domain, respectively. The detailed results for each domain are shown in Supplementary Tables 1–3.

### 3.4 Correlation analysis for KAP

Table 6 presents the Correlations between the KAP domains and their corresponding confidence intervals. The associations among KAP scores were investigated using Spearman’s correlation analysis. The findings showed that all associations were statistically significant at the 0.01 level of significance. In particular, there was a significant positive association between attitude and practice scores (*r* = 0.691, *p* < 0.001), indicating that people with more positive attitudes also practice more consistently. Knowledge and attitude were positively correlated (*r* = 0.659, *p* < 0.001), suggesting that more favorable attitudes are associated with greater knowledge. Likewise, there was a positive correlation between knowledge and practice (*r* = 0.501, *p* < 0.001). Given that attitude shows the largest correlation with both knowledge and practice, these data highlight how intertwined the KAP components are.

**Table 6:** Correlations between the KAP domains.

Variable 1	Variable 2	Correlation	*P*-value	Lower C. I.	Upper C. I.
ATT	KWD	0.659	<0.001*	0.632	0.734
	PRA	0.691	<0.001*	0.641	0.741
KWD	PRA	0.501	<0.001*	0.431	0.574

*Correlation is significant at the 0.01 level.

### 3.5 Linear regression analysis

Supplementary Figures 1–3 show the plots to check the assumption of regression.

The scatterplot in Supplementary Figure 1 shows a clear positive linear trend. The data points are distributed around the fitted regression line without any visible curvature. There is no evidence of systematic deviation suggesting non-linearity.

Supplementary Figure 2 shows the histogram and normal probability plot (P–P plot) of standardized residuals. The plot shows that the residuals appear approximately normally distributed, and the normality assumption is reasonably met.

Supplementary Figure 3 shows the scatterplot of standardized residuals versus predicted values. The plot indicates that the residuals are randomly distributed around the zero line without a systematic curve or visible funnel-shaped pattern, indicating that the assumption of homoscedasticity for standardized residuals is satisfied.

The diagnostic plots indicate that the regression assumptions, namely linearity, normality, and homoscedasticity of residuals, are satisfied.

In addition, the multicollinearity was examined using the Variance Inflation Factor (VIF) for the predictor variables, knowledge and attitude, included in the regression model. The VIF value was 1.345, which is much smaller than commonly accepted thresholds (e.g., VIF < 5 or the more conservative VIF < 10 criterion). A VIF close to 1 indicates minimal correlation among the two predictors, suggesting that multicollinearity is not a concern in the present regression model.

Therefore, the multiple regression model can be considered statistically appropriate, and its parameter estimates are reliable under the classical linear regression framework.

Knowledge and practice were evaluated for their impact on attitude using a multiple linear regression analysis. Detailed results are shown in Table 7. The results show that the value of the variance inflation factor of 1.345 indicates that the predictors’ knowledge and practice domains are not highly correlated, indicating that the multicollinearity problem does not exist. The regression model explained 63.1% of the variance in the attitude domain (*R*^2^ = 0.632, adjusted *R*^2^ = 0.631, *p* < 0.001). The ANOVA findings showed that the whole model was statistically significant (*F* = 357.117, *p* < 0.001), indicating that the combined influence of the predictors, knowledge, and practice on attitude is statistically significant. In addition, the results show that the two domains appeared as significant:

- Knowledge: *t* = 13.041, *p* < 0.001
- Practice: *t* = 13.514, *p* < 0.001

**Table 7:** Analysis of multiple linear regression model.

Variables	Coefficients	Std. error	*T*-value	*P*-value	VIF
(Constant)	0.637	0.093	6.846	<0.001*	
KWD	0.427	0.033	13.041	<0.001*	1.345
PRA	0.399	0.030	13.514	<0.001*	1.345

*R*^2^ = 0.632, adjusted *R*^2^ = 0.631, *F* = 357.117, *P* < 0.001. *The variable is statistically significant at the 0.01 level of significance.

These results indicate that the attitude domain is strongly influenced by both the knowledge and practice domains. These findings imply that attitude is positively influenced by both knowledge and practice.

Safi et al. used Cohen’s f ^2^ to calculate the effect size within a multiple regression model in which the independent variable of interest and the dependent variable are both continuous (43). Cohen’s f ^2^ is commonly presented in a form appropriate for global effect size:


$$f^{2}=\frac{R^{2}}{1-R^{2}}$$

where $R^{2}$ is the coefficient of determination. Effect size measures for f ^2^ are 0.02, 0.15, and 0.35, indicating small, medium, and large effects, respectively.

The effect size f^2^ for the regression models equals 1.717, indicating a large effect of each knowledge and practice domain on the attitude domain. This strong effect reflects a high degree of shared variance among the KAP domains and indicates that the predictors meaningfully contribute to explaining differences in participants’ attitudes.

### 3.6 Hypothesis test for KAP

Regarding the choice of statistical tests, the KAP scores were derived from Likert-scale items, which are ordinal. Given the ordinal measurement level, non-parametric tests were preferred.

Even though Likert scores are sometimes treated as approximately continuous under the central limit theorem, we adopted a conservative approach by using nonparametric tests due to the ordinal structure of the data and the observed non-normality. This ensures robustness of inference without relying on distributional assumptions.

The value 2.5 was selected as the reference point because it represents the theoretical midpoint (median point) of a 4-point Likert scale ranging from 1 to 4. On such a scale, 2.5 lies exactly between the lower response categories (1–2) and the higher response categories (3–4), so it serves as a neutral benchmark to distinguish between below-midpoint and above-midpoint responses. The value 2.5 was used as the null median value in the one-sample signed test.

Table 8 presents the results of the sign test, which was performed to compare the two groups: those scoring less than 2.5 and those scoring greater than 2.5 (the neutral value) on the Knowledge, Attitude, and Practice (KAP) domains.

**Table 8:** Sign test for KAP domains.

Variable	Category	Test value	*P*-value	Effect size
Knowledge score	Group 1 < =2.5	10.22	<0.001*	0.50	Group 2 > 2.5			
	Attitude score	Group 1 < =2.5	18.08	<0.001*	0.88	Group 2 > 2.5			
Practice score		Group 1 < =2.5	16.15	<0.001*	0.79	Group 2 > 2.5			

*The median is significantly greater than 2.5 at the 0.01 level of significance.

The findings indicate that 75% of participants rated the knowledge score above 2.5, 91% rated the attitude above 2.5, and 82% rated the practice domain above 2.5. The test values for knowledge, attitude, and practice domains are 10.22, 18.08, and 16.15, respectively, with a *p*-value less than 0.001. Since the test sign is positive, the mean for each domain is significantly greater than the neutral value of 2.5. This confirms that a statistically significant proportion of respondents (*p* < 0.001 for all domains) rated each domain above 2.5.

Cohen’s effect size measures 0.5, 0.8, and 1.1, indicating small, medium, and large effects, respectively. The effect size ranges between 0.50 and 0.88, which, according to Cohen’s criteria for nonparametric tests, represents a medium to large effect. This suggests a meaningful difference in KAP domains between the two groups, less than and greater than the neutral value of 2.5. These findings reflect a widespread favorable KAP toward the use of medicinal plants in the community. The detailed results for each domain are shown in Supplementary Tables 4–6.

### 3.7 Hypothesis test for KAP with sociodemographic variables

Table 9 presents the results of the hypothesis test examining the effect of gender on KAP domains across sociodemographic variables. The detailed results for each domain are shown in Supplementary Tables 7–11.

**Table 9:** Independent samples test for sociodemographic variables.

Domain	Gender		Age		Education		Employment		Herbal medicine	
	*P*-value	Effect size	*P*-value	Effect size	*P*-value	Effect size	*P*-value	Effect size	*P*-value	Effect size
Knowledge	<0.001*	0.37	0.009*	0.023	<0.001*	0.043	<0.001*	0.058	<0.001*	0.072
Attitude	<0.001*	0.41	<0.001*	0.065	<0.001*	0.071	<0.001*	0.094	<0.001*	0.132
Practice	<0.001*	0.31	0.001*	0.035	<0.001*	0.047	<0.001*	0.065	<0.001*	0.148

*The mean difference is significant at the 0.01 level of significance.

The effect size for the Mann–Whitney test is calculated as Cohen’s r (small ≈ 0.10, medium ≈ 0.30, large ≈ 0.50), in accordance with conventional guidelines for *r*.


$$r=\frac{Z}{\sqrt{N}}$$

where *Z* is the standardized test statistic from the Mann–Whitney U test and *N* is the total sample size.

The effect size for the Kruskal-Wallis test is calculated as Eta-Squared, with measures of 0.06, 0.14, and 0.23, indicating small, medium, and large effects, respectively.

For gender, the Mann–Whitney test shows statistically significant differences between male and female participants across all three domains (*p*-value < 0.001). The results show that the mean rank for males is higher than that for females in each KAP domain. With effect sizes ranging from 0.31 to 0.41, which are considered medium according to Cohen’s guidelines, it is evident that gender moderately influences knowledge, attitudes, and practices in the context studied.

The Kruskal-Wallis test is used to examine the effect of age on KAP domains for the other sociodemographic variables.

For age, the results show that there is a statistically significant difference in knowledge, attitude, and practice scores among the age groups (*p* = 0.009, <0.001, and 0.001, respectively), with an Eta squared value of 0.023, 0.065, and 0.035, respectively, suggesting a small to moderate effect size. This indicates that while the result is significant, the practical difference for knowledge is minimal. Middle-aged participants (35–54) scored higher in knowledge compared to the youngest (18–24) and oldest (55–64) groups.

In addition, the results suggest that age has a clear impact on attitudes, with individuals aged 25–44 displaying the most favorable attitudes, while younger participants (18–24) exhibit the least favorable attitudes. Furthermore, for the practice domain, the result indicates a small-to-moderate effect size. This implies that practice behaviors vary with age, with middle-aged adults engaging more in the desired practices compared to younger and older groups. Overall, age has the strongest influence on attitude, followed by practice and knowledge. Middle-aged groups (25–54 years) generally demonstrate more favorable outcomes across all three domains, while younger (18–24) and older (55–64) groups tend to have lower scores.

For educational levels, the results show a statistically significant difference in knowledge scores (*p* < 0.001). Individuals with less than a high school education had the highest mean rank, suggesting greater knowledge in this group, an unexpected result that may warrant further exploration.

However, the effect size (0.043) is small, indicating that although statistically significant, the practical difference is limited. In addition, the result indicates a statistically significant difference in attitude scores across education levels (*p* < 0.001), with less than high school and master’s degree holders scoring highest, while PhD holders scored lowest. The effect size (0.071) indicates a small-to-moderate effect, suggesting a more notable, though still modest, practical impact. Furthermore, practice scores significantly differ by education level (*p* < 0.001).

Also, the less-than-high-school group had the highest rank, while PhD holders had the lowest. The effect size of 0.047 indicates a small practical difference. Overall, the Kruskal-Wallis test indicated statistically significant differences across educational levels for knowledge, attitude, and practice. Despite statistical significance (*p* < 0.001 in all cases), the effect sizes (0.043–0.071) suggest practical significance ranging from small to small-moderate. Interestingly, lower education levels (Less than high school) often had higher scores, possibly due to unique background, cultural, or sampling factors that are worth further investigation.

For employment categories, the results show that Knowledge scores varied significantly across employment groups (*p* < 0.001), with employed individuals having the highest mean rank, indicating greater knowledge levels, while students had the lowest scores. The effect size of 0.058 suggests a small to moderate practical difference.

Attitude scores also showed significant differences by employment status, with employed individuals scoring the highest and unemployed individuals the lowest. The effect size of 0.094 indicates a moderate practical difference. Practice scores also differed significantly (p < 0.001), with employed individuals ranking highest and students lowest. The effect size of 0.065 suggests a small to moderate practical difference.

Overall, employed participants consistently scored highest across all domains. Students and unemployed participants generally scored lower. The effect sizes, ranging from 0.058 to 0.094, indicate small to moderate practical significance. These findings imply that employment status may play a meaningful role in shaping individuals’ knowledge, attitudes, and practices, potentially reflecting differences in exposure, responsibility, or access to information and resources.

Regarding herbal medicine intake, the results show that Knowledge scores varied significantly by how often individuals took herbal medicine. Those who “Often” or “Always” used herbal medicine had higher scores than those who “Never” or “Rarely” used it. The effect size (η^2^ = 0.072) indicates a moderate practical effect.

Attitude scores also showed significant variation with usage frequency. Individuals who “Often” or “Always” used herbal medicine had more favorable attitudes. The effect size (η^2^ = 0.132) suggests a moderate practical significance. Practice scores exhibited the most substantial differences across groups. Frequent users of herbal medicine had significantly higher practice scores. The effect size (η^2^ = 0.148) indicates a large effect, highlighting a meaningful association between herbal medicine use frequency and related practices.

In summary, the frequency of herbal medicine intake is significantly associated with differences in knowledge, attitude, and practice scores. Participants who frequently use herbal medicine (“Often” and “Always”) consistently report higher scores across all domains. The largest effects are observed in the practice domain (η^2^ = 0.148) and attitude domain (η^2^ = 0.132), suggesting substantial practical differences. These findings indicate that increased engagement with herbal medicine is linked to greater knowledge, more positive attitudes, and more active related practices.

### 3.8 Summary of findings in relation to study hypotheses

The statistical findings support the study hypotheses. First, the majority of participants demonstrated knowledge scores above the midpoint of the scale, indicating moderate to high levels of knowledge about medicinal plants. Second, significant positive correlations were observed among knowledge, attitudes, and practices (*p* < 0.001), confirming the proposed interrelationships among the KAP domains. Third, sociodemographic variables—including age, gender, education level, employment status, and frequency of herbal medicine use—were significantly associated with KAP scores, supporting the hypothesis that demographic factors influence medicinal plant–related knowledge and behavior.

## 4 Discussion

Building on the statistical findings presented above, this section interprets the results within broader cultural, regional, and methodological contexts. The present study provides a comprehensive assessment of KAP regarding MP use in the UAE. Our findings reveal important insights into public perceptions and behaviors, aligning with and diverging from previous research across various cultural and regional contexts. Regional and global evidence also illustrates growing scientific and clinical interest in plant-derived therapeutics, including systematic reviews of polyphenol interventions for metabolic disease and reviews of key medicinal plants such as *Nigella sativa* and *Ziziphus lotus* (11, 13, 44).

An important methodological consideration concerns internal consistency across KAP domains. Although the overall instrument demonstrated strong reliability (*α* = 0.870), variability was observed at the subdomain level. The initial knowledge domain alpha (α = 0.502) improved following item-level refinement, suggesting potential heterogeneity among the knowledge statements. Additionally, the practice domain demonstrated lower internal consistency (α = 0.592), which may reflect the limited number of Likert-scale items and the multidimensional nature of behavioral constructs. Behavioral measures often exhibit lower internal consistency compared to attitudinal scales due to situational variability and context-dependent responses, and values around 0.70 are generally considered acceptable in social science research (45). Similar variability in knowledge assessment has been reported in other community-based studies (27), underscoring the importance of culturally adapted instruments. These findings suggest that future studies may benefit from expanding the number of practice items or applying confirmatory factor analysis to strengthen construct stability.

Moreover, the current findings indicate that age, gender, education, employment status, and MP usage frequency significantly influence KAP. Males exhibited higher KAP scores than females, contrary to Aljawarneh et al., who found no significant gender differences in complementary alternative medicine (CAM) use in the UAE (33). This difference may stem from differing sample compositions or cultural perceptions of MP.

Additionally, age played a significant role, with middle-aged individuals (25–54 years) exhibiting more favorable KAP scores than both younger (18–24) and older (55–64) groups. This aligns with Issa and Basheti, who found that middle-aged Jordanians had more positive attitudes toward herbal medicine due to increased exposure and health concerns (25). However, Chowdhury et al. reported greater knowledge among older rural Indian populations, indicating that cultural and educational contexts outline age-related differences (46).

Furthermore, education and employment status also influenced KAP, with employed individuals scoring higher than students and unemployed respondents. An unexpected finding was that participants with lower formal education levels demonstrated comparatively higher knowledge scores than those with advanced degrees. This result should be interpreted cautiously. It may reflect cultural patterns of traditional knowledge transmission within families rather than formal health literacy. Additionally, sampling composition, self-reported measures, and potential response bias may have influenced this outcome. Given the relatively small proportion of participants in certain education categories, these subgroup differences may not fully represent the broader population. Future research using stratified sampling and mixed-methods approaches would help clarify whether this pattern reflects genuine cultural knowledge dynamics or methodological effects, a finding that differs from Pungong et al., who found that higher education was linked to better knowledge in Cameroon (26). This difference may reflect traditional knowledge transmission among less formally educated groups in the UAE, necessitating further qualitative investigation.

The strong positive correlations observed between knowledge and attitude (*r* = 0.659), knowledge and practice (*r* = 0.501), and attitude and practice (*r* = 0.691) highlight the interconnected nature of these domains. Additionally, knowledge and practice together explained a substantial proportion of the variance in attitude (*R*^2^ = 0.631), indicating that informational exposure and behavioral engagement are closely aligned. Nevertheless, given the cross-sectional design, these findings should be interpreted as associative rather than causal relationships. The results support the notion that knowledge, perceptions, and practices toward medicinal plants function as mutually reinforcing components within the sociocultural context of the UAE, suggesting that Higher levels of knowledge were significantly associated with more positive attitudes and practices. These findings are consistent with Zhou et al., who highlighted that education and awareness campaigns could improve perceptions of herbal medicine safety and efficacy (28).

The relatively high proportions of participants scoring above the scale midpoint for knowledge (75%), attitude (91%), and practice (82%) suggest generally favorable perceptions of medicinal plant use in the UAE. However, these patterns should not be interpreted as evidence of uniformly comprehensive understanding. The particularly high attitude scores may reflect cultural normalization of herbal remedies, which are often embedded within family traditions and regional practices. In contrast, the more moderate knowledge scores indicate that positive perceptions may coexist with partial or perception-based understanding rather than fully evidence-based awareness. Similar trends have been observed in regional studies in Saudi Arabia and Jordan, where widespread acceptance of herbal medicine did not necessarily correspond with detailed safety awareness. Therefore, while the numerical findings indicate strong engagement with medicinal plants, they likely represent a combination of cultural familiarity, accessibility, and perceived safety rather than strictly formal health literacy.

However, similar to Aljawarneh et al., we found that frequent MP users had significantly higher KAP scores, emphasizing the importance of personal experience in shaping perceptions (33).

Furthermore, the strong link between MP use frequency and KAP indicates that targeted educational efforts could enhance safe and effective use. Policymakers should consider integrating awareness of traditional medicine into public health campaigns, as recommended by Zhou et al. (28). Additionally, the unexpected education-level findings emphasize the importance of culturally sensitive KAP assessments to prevent misinterpretation of traditional knowledge systems.

Beyond descriptive statistics, this study makes a unique contribution to the regional literature in several ways. First, it represents the first dedicated assessment of medicinal plant–specific KAP in the UAE, distinguishing plant-based practices from broader complementary and alternative medicine categories. Second, by statistically modeling the interrelationships among knowledge, attitudes, and practices, the study moves beyond prevalence reporting to examine structural associations within the KAP framework. Third, the identification of sociodemographic patterns within a culturally diverse Gulf population provides context-specific insight not previously documented in UAE-based research. Collectively, these elements position the study as both an empirical contribution to KAP methodology and a regionally relevant foundation for evidence-based public health planning in the Gulf.

## 5 Limitations and future research

This study has several limitations. First, the cross-sectional design prevents causal inference about the relationships among knowledge, attitudes, and practices. Observed relationships should therefore be interpreted as associative rather than causal.

Second, data were collected via an online self-administered survey, which may have introduced selection bias by favoring younger, more technologically engaged participants. This is reflected in the sample’s demographic composition, where the majority were young adults (58% aged 18–24 years) and highly educated (56.5% with a Bachelor’s degree). Such demographic concentration may limit the generalizability of the findings to older individuals and those with lower educational attainment. Future studies should employ stratified or probability-based sampling approaches to ensure broader demographic representation.

Third, reliance on self-reported responses may introduce social desirability bias, potentially inflating reported knowledge levels or positive attitudes toward medicinal plants.

Fourth, although the overall instrument demonstrated acceptable internal consistency, variability was observed across subdomains, particularly within the practice domain. The practice subscale’s lower reliability may reflect the limited number of items and the inherent variability of behavioral constructs.

Fifth, structural validity was not formally evaluated using exploratory or confirmatory factor analysis. While internal consistency was assessed, the absence of factor-analytic validation limits conclusions regarding construct dimensionality. Future research should incorporate factor analysis to enhance the instrument’s psychometric robustness.

Finally, the absence of qualitative data limits deeper exploration of unexpected subgroup findings, particularly the higher knowledge scores observed among participants with lower levels of formal education. Mixed-method approaches could provide richer contextual insight. Additionally, as the questionnaire was administered in English, interpretation variability among non-native English speakers cannot be excluded. Future studies may benefit from validated bilingual instruments to ensure linguistic and cultural equivalence.

Comparative research across Gulf Cooperation Council countries may further clarify regional similarities and differences in medicinal plant–related knowledge, attitudes, and practices.

## 6 Conclusion

This study, to our knowledge, represents the first dedicated assessment of knowledge, attitudes, and practices related to medicinal plant use in the United Arab Emirates. By distinguishing medicinal plants from broader complementary and alternative medicine constructs and by integrating analytical modeling into the KAP framework, the study provides context-specific, methodologically strengthened insight into public perceptions and behavioral patterns in the UAE.

The findings indicate generally favorable engagement with medicinal plants while revealing meaningful sociodemographic variations and areas requiring targeted educational attention. The strong associations observed among knowledge, attitudes, and practices underscore the importance of structured, evidence-based awareness initiatives rather than relying solely on culturally transmitted perceptions.

From a public health and regulatory perspective, the results support several targeted actions: incorporating evidence-based information on medicinal plants into national health awareness campaigns; integrating herbal medicine safety, drug–herb interaction education, and regulatory guidance into healthcare and medical curricula; strengthening labeling standards and post-market surveillance of herbal products; and encouraging healthcare professionals to routinely inquire about herbal medicine use during clinical consultations.

Collectively, these measures may promote safer, more informed, and culturally sensitive integration of medicinal plant use within the UAE healthcare framework.

## Data Availability

The original contributions presented in the study are included in the article/Supplementary material, further inquiries can be directed to the corresponding author.

## References

[1] Jamshidi-Kia F Lorigooini Z Amini-Khoei H. Medicinal plants: past history and future perspective. J Herbmed Pharmacol. (2018) 7:1–7. doi: 10.15171/jhp.2018.01

[2] Sen S Chakraborty R. Revival, modernization and integration of Indian traditional herbal medicine in clinical practice: importance, challenges and future. J Tradit Complement Med. (2017) 7:234–44. doi: 10.1016/j.jtcme.2016.05.006, 28417092 PMC5388083

[3] Khadka D Dhamala MK Li F Aryal PC Magar PR Bhatta S . The use of medicinal plants to prevent COVID-19 in Nepal. J Ethnobiol Ethnomed. (2021) 17:26. doi: 10.1186/s13002-021-00449-w, 33832492 PMC8027983

[4] Chaachouay N Zidane L. Plant-derived natural products: a source for drug discovery and development. DDC. (2024) 3:184–207. doi: 10.3390/ddc3010011

[5] Najmi A Javed SA Al Bratty M Alhazmi HA. Modern approaches in the discovery and development of plant-based natural products and their analogues as potential therapeutic agents. Molecules. (2022) 27:349. doi: 10.3390/molecules27020349, 35056662 PMC8779633

[6] Wang X Anwar T Qureshi H El-Beltagi HS Sehar Z Solieva D . Plant-based traditional remedies and their role in public health: ethnomedicinal perspectives for a growing population. J Health Popul Nutr. (2025) 44:300. doi: 10.1186/s41043-025-01036-5, 40836265 PMC12369213

[7] Tran N Pham B Le L. Bioactive compounds in anti-diabetic plants: from herbal medicine to modern drug discovery. Biology. (2020) 9:252. doi: 10.3390/biology9090252, 32872226 PMC7563488

[8] Figueroa C Echeverría G Villarreal G Martínez X Ferreccio C Rigotti A. Introducing plant-based Mediterranean diet as a lifestyle medicine approach in Latin America: opportunities within the Chilean context. Front Nutr. (2021) 8:8. doi: 10.3389/fnut.2021.680452, 34249989 PMC8266999

[9] Craig WJ Mangels AR Fresán U Marsh K Miles FL Saunders AV . The safe and effective use of plant-based diets with guidelines for health professionals. Nutrients. (2021) 13:4144. doi: 10.3390/nu13114144, 34836399 PMC8623061

[10] Bedir AS Almasri RS Azar YO Elnady RE Al Raish SM. Exploring the therapeutic potential of Allium cepa and *Allium sativum* extracts: current strategies, emerging applications, and sustainability utilization. Biology. (2025) 14:1088. doi: 10.3390/biology14081088, 40906415 PMC12383551

[11] Bedir AS Almasri RS Al Raish SM. Therapeutic efficacy of Nigella sativa and *Ziziphus lotus*: sustainable strategies for diabetes, antimicrobial resistance, and health treatment. Front Nutr. (2025) 12:12. doi: 10.3389/fnut.2025.1592423, 40969600 PMC12442494

[12] Al Raish SM Almasri RS Bedir AS Elkahwagy AA. Phytochemical composition, bioactive compounds, and antidiabetic potential of four medicinal plants native to the UAE: *Capparis spinosa*, *Citrullus colocynthis*, Morus alba, and Rhazya stricta. Biology. (2025) 14:1146. doi: 10.3390/biology14091146, 41007291 PMC12467449

[13] Ranneh Y Bedir AS Abu-Elsaoud AM Al Raish S. Polyphenol intervention ameliorates non-alcoholic fatty liver disease: an updated comprehensive systematic review. Nutrients. (2024) 16:4150. doi: 10.3390/nu16234150 39683546 PMC11644642

[14] Arjona-García C Blancas J Beltrán-Rodríguez L López Binnqüist C Colín Bahena H Moreno-Calles AI . How does urbanization affect perceptions and traditional knowledge of medicinal plants? J Ethnobiology Ethnomedicine. (2021) 17:48. doi: 10.1186/s13002-021-00473-w, 34344391 PMC8330055

[15] Obradović S Stojanović V Milić D. The importance of understanding local community attitudes and perceptions regarding nature conservation. Wetlands. (2022) 43:2. doi: 10.1007/s13157-022-01652-5

[16] Okaiyeto K Oguntibeju OO. African herbal medicines: adverse effects and cytotoxic potentials with different therapeutic applications. Int J Environ Res Public Health. (2021) 18:5988. doi: 10.3390/ijerph18115988, 34199632 PMC8199769

[17] Salm S Rutz J van den Akker M Blaheta RA Bachmeier BE. Current state of research on the clinical benefits of herbal medicines for non-life-threatening ailments. Front Pharmacol. (2023) 14:14. doi: 10.3389/fphar.2023.1234701, 37841934 PMC10569491

[18] Wang H Chen Y Wang L Liu Q Yang S Wang C. Advancing herbal medicine: enhancing product quality and safety through robust quality control practices. Front Pharmacol. (2023) 14:14. doi: 10.3389/fphar.2023.1265178, 37818188 PMC10561302

[19] Dores AR Peixoto M Castro M Sá C Carvalho IP Martins A . Knowledge and beliefs about herb/supplement consumption and herb/supplement–drug interactions among the general population, including healthcare professionals and pharmacists: a systematic review and guidelines for a smart decision system. Nutrients. (2023) 15:2298. doi: 10.3390/nu15102298, 37242184 PMC10220613

[20] Gamil NM Elsayed HA Hamed RM Salah ET Ahmed AM Mostafa HA . Insights from herb interactions studies: a foundational report for integrative medicine. Futur J Pharm Sci. (2025) 11:46. doi: 10.1186/s43094-025-00794-7

[21] Ghassab-Abdollahi N Ghorbani Z Kheirollahi N Nadrian H Hashemiparast M. Exploring the reasons for self-administration medication errors among illiterate and low-literate community-dwelling older adults with polypharmacy: a qualitative study. BMC Geriatr. (2024) 24:1–14. doi: 10.1186/s12877-024-05595-w, 39702061 PMC11658105

[22] Chunarkar-Patil P Kaleem M Mishra R Ray S Ahmad A Verma D . Anticancer drug discovery based on natural products: from computational approaches to clinical studies. Biomedicine. (2024) 12:201. doi: 10.3390/biomedicines12010201, 38255306 PMC10813144

[23] Surigao del Norte State University Philippines Elicano LML. Knowledge, attitude, and practices (KAP) in complementary and alternative medicine (CAM) among selected Asian countries: a literature review. ijcsrr. (2024) 7:707–714. doi: 10.47191/ijcsrr/V7-i6-22

[24] Jia H Li C Li Z Zhao Y Chen Z Duan J . Knowledge, attitudes, and practices regarding food-medicine dual-purpose substances among adults in China: a cross-sectional online survey. BMC Complement Med Ther. (2025) 25:76. doi: 10.1186/s12906-025-04822-0, 39994653 PMC11854228

[25] Issa RA Basheti IA. Herbal medicine use by people in Jordan: exploring believes and knowledge of herbalists and their customers. J Biol Sci. (2017) 17:400–9. doi: 10.3923/jbs.2017.400.409

[26] Pungong CS Langsi DJ Fotso B. Knowledge, aptitudes and practice on traditional medicinal plant use in Bafanji Village (Ngoketunjia division, north west region, Cameroon). J Biotechnol. (2023) 2:1–21. doi: 10.36108/jbt/3202.20.0110

[27] Wassie SM Aragie LL Taye BW Mekonnen LB. Knowledge, attitude, and utilization of traditional medicine among the communities of Merawi town, Northwest Ethiopia: a cross-sectional study. Evid Based Complement Alternat Med. (2015) 2015:1–7. doi: 10.1155/2015/138073, 26508974 PMC4609866

[28] Zhou X Li CG Chang D Bensoussan A. Current status and major challenges to the safety and efficacy presented by Chinese herbal medicine. Medicine. (2019) 6:14. doi: 10.3390/medicines6010014, 30669335 PMC6473719

[29] Alhomoud FK. Manuscript title: regulatory and implications of complementary and alternative medicine (CAM) use for infection prevention: insights from the Gulf cooperation council (GCC) countries. Risk Manag Healthc Policy. (2025) 18:2081–90. doi: 10.2147/RMHP.S522405, 40589540 PMC12206891

[30] Al-Worafi YM. "Complementary and alternative medicine (CAM) in Saudi Arabia". In: Handbook of Complementary, Alternative, and Integrative Medicine. Boca Raton, FL: CRC Press (2025)

[31] Alsanad SM. A comprehensive look at complementary and alternative medicine (CAM) in Saudi Arabia: a Meta-analysis study. Health Sci Reports. (2025) 8:e70491. doi: 10.1002/hsr2.70491, 40256149 PMC12006920

[32] Al-Worafi YM Othman G Alakhali KM Dhabali AA. "Complementary and alternative medicine (CAM) in Yemen". In: Handbook of Complementary, Alternative, and Integrative Medicine. Boca Raton, FL: CRC Press (2025)

[33] Aljawarneh Y Rajab L Alzeyoudi A Alzeyoudi A Ibrahim A Alnaqbi N . Knowledge, attitude, and practice toward complementary alternative medicine in the UAE: a cross- sectional study. Eur J Integr Med. (2023) 64:102310. doi: 10.1016/j.eujim.2023.102310

[34] Al Raish S Platat C.The first AAU international conference on pharmacy and biomedical sciences. BMC Proc. (2023) 17:25. doi: 10.1186/s12919-023-00276-9, 37794446 PMC10552187

[35] Alhumaid MM Alobaid MA Said MA. Patterns, motivations, and determinants of dietary supplement use among physically active adults in eastern Saudi Arabia: a cross-sectional survey. Front Public Health. (2026):14. doi: 10.3389/fpubh.2026.1734477, 41657692 PMC12872503

[36] Pennino F Maccauro ML Sorrentino M Gioia M Riello S Messineo G . Insights from a cross-sectional study on knowledge, attitudes and Behaviors concerning antibiotic use in a large metropolitan area: implications for public health and policy interventions. Antibiotics. (2023) 12:1476. doi: 10.3390/antibiotics12101476, 37887177 PMC10603846

[37] Thangavelu PD Janakiraman B Pawar R Shingare PH Bhosale S Souza RD . Understanding, being, and doing of bioethics; a state-level cross-sectional study of knowledge, attitude, and practice among healthcare professionals. BMC Med Ethics. (2024) 25:30. doi: 10.1186/s12910-024-01028-w, 38500167 PMC10949768

[38] Sheehama WLN Singh T. Food safety in informal markets: how knowledge and attitudes influence vendor practices in Namibia. Int J Environ Res Public Health. (2025) 22:631. doi: 10.3390/ijerph22040631, 40283854 PMC12026613

[39] Nguyen PH Tran VD Pham DT Dao TNP Dewey RS. Use of and attitudes towards herbal medicine during the COVID-19 pandemic: a cross-sectional study in Vietnam. Eur J Integr Med. (2021) 44:101328. doi: 10.1016/j.eujim.2021.101328, 365s70027 36570027 PMC9760728

[40] Zaidi SF Saeed SA Khan MA Khan A Hazazi Y Otayn M . Public knowledge, attitudes, and practices towards herbal medicines; a cross-sectional study in Western Saudi Arabia. BMC Complement Med Ther. (2022) 22:326. doi: 10.1186/s12906-022-03783-y, 36482398 PMC9733054

[41] Soltanipour S Keihanian F Saeidinia A. Knowledge, attitude and practice of physicians towards herbal remedies in Rasht, north of Iran. Medicine. (2022) 101:e31762. doi: 10.1097/MD.0000000000031762, 36451387 PMC9704890

[42] Al Akeel M Al Ghamdi W Al Habib S Koshm M Al OF. Herbal medicines: Saudi population knowledge, attitude, and practice at a glance. J Family Med Prim Care. (2018) 7:865–75. doi: 10.4103/jfmpc.jfmpc_315_17, 30598925 PMC6259532

[43] Safi SK Dorgham MM Allaloul SA. Impact of senior management’s financial intelligence on the financial performance of banks and insurance companies in the Gaza strip. Discov Sustain. (2024) 5:84. doi: 10.1007/s43621-024-00232-3

[44] Latif R Nawaz T. Medicinal plants and human health: a comprehensive review of bioactive compounds, therapeutic effects, and applications. Phytochem Rev. (2025). doi: 10.1007/s11101-025-10194-7

[45] Hussey I Alsalti T Bosco F Elson M Arslan R. An aberrant abundance of Cronbach’s alpha values at 70. Adv Methods Pract Psychol Sci. (2025) 8:25152459241287. doi: 10.1177/25152459241287123

[46] Chowdhury CR Dey Chowdhury A Khijmatgar S Markus AF. Level of oral cancer awareness among Indian rural population: a possible research model using knowledge, attitude and practice (KAP) intervention and its utilisation in low resource settings of LMICs. J Oral Biol Craniofac Res. (2022) 12:154–60. doi: 10.1016/j.jobcr.2021.10.008, 34824968 PMC8604810

[47] Hair JF Jr Black WC Babin BJ Anderson RE. Multivariate data analysis. 7th ed. Upper Saddle River (NJ): Prentice Hall. (2010) Available from: https://openlibrary.org/books/OL22691711M/Multivariate_data_analysis, 34824968

