# Supplementary material for: Insights into knowledge, attitudes, and practices of medicinal plant use in the United Arab Emirates: a cross-sectional study
Source: Front Nutr. 2026 Mar 16;13:1755440. doi: 10.3389/fnut.2026.1755440 (PMC13033477; doi:10.3389/fnut.2026.1755440)
Supplement: Supplementary file 1 [file Data_Sheet_1.pdf]

Table A1. Descriptive Statistics for Knowledge Domain

Knowledge Variables Questions	Mean	SDV
Herbal medicines are made from plant sources	3.44	0.59
Herbal medicines can prevent all diseases	2.80	0.89
Herbal medicines can cure all diseases	2.60	0.89
Herbal medicine is always safe	2.71	0.92
Overuse of herbal medicine can cause adverse effects	3.17	0.77
Herbal medicines can be taken with conventional or allopathic medicines	2.91	0.79
Herbal medicines don't expire	2.31	0.94
**Knowledge Score**	**2.85**	**0.49**

Table A2. Descriptive Statistics for Attitude Domain

Attitude Variables Questions	Mean	SDV
Herbal medicines are safe because they are made from natural ingredients	3.10	0.77
Herbal medicines are better for me than conventional or allopathic medicines	2.96	0.84
A lot of the health claims made by the manufacturers of herbal medicines are unproven	3.00	0.78
I prefer herbal medicines because they are cheap and easily available	2.84	0.89
It is important to talk to a medical doctor or herbal doctor, or pharmacist before using herbal medicines	3.30	0.74
To what extent do you trust the published information on traditional recipes in commercial channels?	2.91	0.85
The effect of herbal medicine is usually due to its placebo effect	2.84	0.83
Herbal medicine should be researched and evaluated more by universities.	3.37	0.70
If an herbal medicine has an equal effect to a chemical drug, I choose the herbal medicine.	3.29	0.76
What is your opinion on putting herbal medicine education in continuous medical education programs?	3.34	0.73
**Attitude score**	**3.09**	**0.46**

Table A3. Descriptive Statistics for Practice Domain

Practice Variables Questions	Mean	SDV
When I get sick, I first take Herbal medicines to help me get better	3.02	0.88
I take Herbal medicines according to the instructions on the label	3.17	0.75
I always look at the expiry date of Herbal medicines before taking them	3.25	0.76
I advise others to take Herbal medicines whenever they have problems	3.01	0.81
**Practice Score**	**3.11**	**0.54**

Table A4. Sign Test for the Knowledge Domain

Variable	Category	Test Value	P-value	Effect Size
Herbal medicines are made from plant sources	Group 1<=2.5<br>Group 2 >2.5	18.64	<0.001*	0.91
Herbal medicines can prevent all diseases	Group 1<=2.5<br>Group 2 >2.5	4.55	<0.001*	0.22
Herbal medicines can cure all diseases	Group 1<=2.5<br>Group 2 >2.5	0.15	0.833	0.01
Herbal medicine is always safe	Group 1<=2.5<br>Group 2 >2.5	2.59	<0.001*	0.13
Overuse of herbal medicine can cause adverse effects	Group 1<=2.5<br>Group 2 >2.5	13.55	<0.001*	0.66
Herbal medicines can be taken with conventional or allopathic medicines	Group 1<=2.5<br>Group 2 >2.5	8.95	<0.001*	0.44
Herbal medicines don't expire	Group 1<=2.5<br>Group 2 >2.5	4.55	<0.001*	0.22

* The median is significantly greater than 2.5

Table A5. Sign Test for the Attitude Domain

Variable	Category	Test Value	P-value	Effect Size
Herbal medicines are safe because they are made from natural ingredients	Group 1<=2.5<br>Group 2 >2.5	12.28	<0.001*	0.60
Herbal medicines are better for me than conventional or allopathic medicines	Group 1<=2.5<br>Group 2 >2.5	8.66	<0.001*	0.42
A lot of the health claims made by the manufacturers of herbal medicines are unproven	Group 1<=2.5<br>Group 2 >2.5	9.64	0.833	0.47
I prefer herbal medicines because they are cheap and easily available	Group 1<=2.5<br>Group 2 >2.5	5.53	<0.001*	0.27
It is important to talk to a medical doctor or herbal doctor, or pharmacist before using herbal medicines	Group 1<=2.5<br>Group 2 >2.5	14.72	<0.001*	0.72
To what extent do you trust the published information on traditional recipes in commercial channels?	Group 1<=2.5<br>Group 2 >2.5	8.76	<0.001*	0.43
The effect of herbal medicine is usually due to its placebo effect	Group 1<=2.5<br>Group 2 >2.5	6.99	<0.001*	0.34
Herbal medicine should be researched and evaluated more by universities.	Group 1<=2.5<br>Group 2 >2.5	16.19	<0.001*	0.79
If an herbal medicine has an equal effect to a chemical drug, I choose the herbal medicine.	Group 1<=2.5<br>Group 2 >2.5	14.72	<0.001*	0.72
What is your opinion on putting herbal medicine education in continuous medical education programs?	Group 1<=2.5<br>Group 2 >2.5	15.80	<0.001*	0.77

* The median is significantly greater than 2.5

Table A6. Sign Test for the Practice Domain

Variable	Category	Test Value	P-value	Effect Size
When I get sick, I first take Herbal medicines to help me get better	Group 1<=2.5<br>Group 2 >2.5	9.44	<0.001*	0.46
I take Herbal medicines according to the instructions on the label	Group 1<=2.5<br>Group 2 >2.5	13.74	<0.001*	0.67
I always look at the expiry date of Herbal medicines before taking them	Group 1<=2.5<br>Group 2 >2.5	14.04	0.833	0.69
I advise others to take Herbal medicines whenever they have problems	Group 1<=2.5<br>Group 2 >2.5	10.61	<0.001*	0.52

* The median is significantly greater than 2.5

Table A7. Independent Samples Mann-Whitney Test for gender

Domain	Mean Rank		Test Value	P-value	Effect Size
	Male	Female			
Knowledge	248.38	170.99	6.580	<0.001*	0.37
Attitude	252.76	166.65	7.310	<0.001*	0.41
Practice	242.23	177.09	5.582	<0.001*	0.31

* The mean difference is significant at the 0.01 level of significance.

Table A8. Independent Samples Kruskal-Wallis Test for Age

Domain	Mean Rank					Test Value	P-value	Effect Size
	18-24 years	25-34 years	35-44 years	45-54 years	55-64 years			
Knowledge	192.95	228.83	239.40	247.43	159.20	13.572	0.009*	0.023
Attitude	182.31	247.07	253.60	244.25	203.00	30.735	<0.001*	0.065
Practice	189.07	238.78	241.94	238.11	183.20	18.319	0.001*	0.035

* The mean difference is significant at the 0.01 level of significance.

Table A9. Independent Samples Kruskal-Wallis Test for Education

Domain	Mean Rank					Test Value	P-value	Effect Size
	< high school	High school	Bachelor	Master	PhD			
Knowledge	332.33	174.11	223.05	230.00	197.00	21.885	<0.001*	0.043
Attitude	316.75	165.55	227.81	242.26	121.50	33.359	<0.001*	0.071
Practice	281.67	176.02	228.25	213.74	88.90	23.586	<0.001*	0.047

* The mean difference is significant at the 0.01 level of significance.

Table A10. Independent Samples Kruskal-Wallis Test for Employment

Domain	Mean Rank				Test Value	P-value	Effect Size
	Employed	Retired	Student	Unemployed			
Knowledge	243.66	207.38	177.42	202.04	27.005	<0.001*	0.058
Attitude	253.54	201.56	176.29	172.31	41.813	<0.001*	0.094
Practice	246.01	190.38	177.98	197.05	29.815	<0.001*	0.065

* The mean difference is significant at the 0.01 level of significance.

Table A11. Independent Samples Kruskal-Wallis Test for Herbal Medicine Intake

Domain	Mean Rank				Test Value	P-value	Effect Size
	Never	Rarely	Often	Always			
Knowledge	177.20	175.48	245.89	233.59	32.638	<0.001*	0.072
Attitude	162.16	167.92	257.21	243.90	57.474	<0.001*	0.132
Practice	157.99	167.91	260.43	241.55	64.384	<0.001*	0.148

* The mean difference is significant at the 0.01 level of significance.

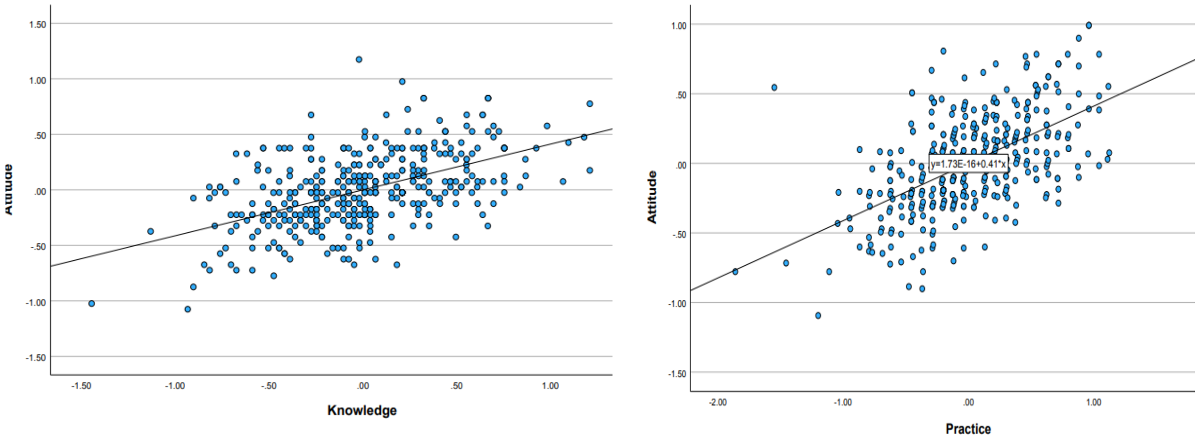

Figure 1: Linearity Assumption

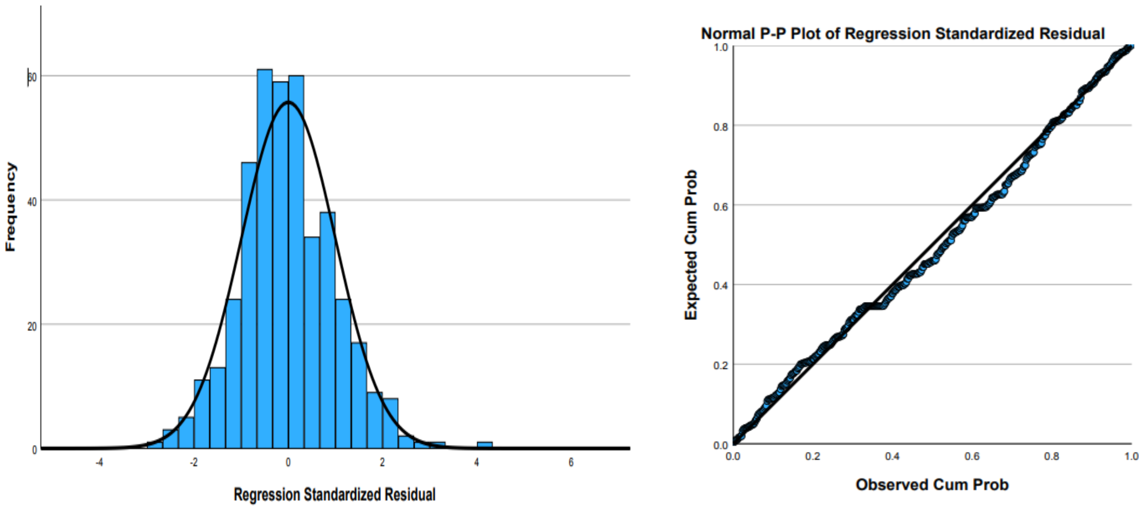

Figure 2: Normality Assumption **for the Standardized Residuals**

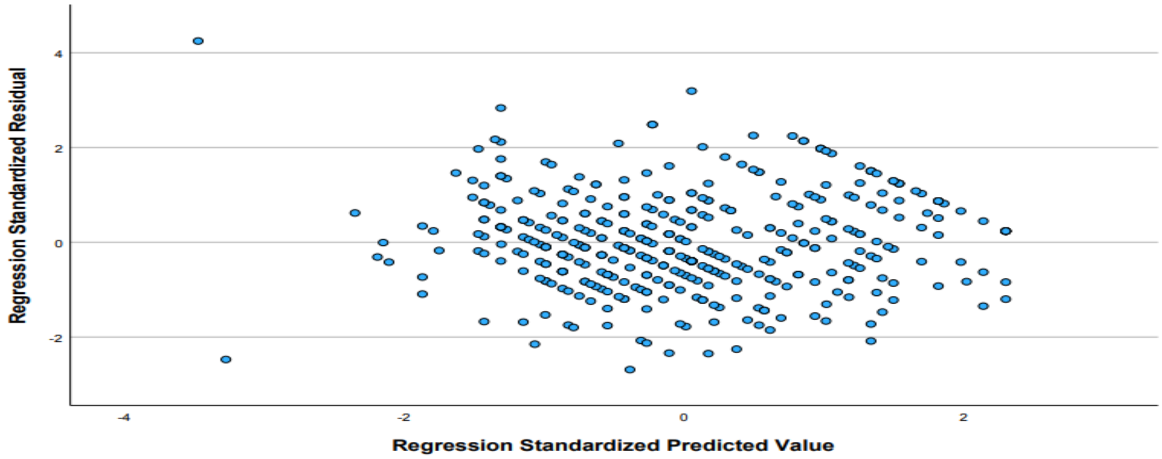

Figure 3: Homoscedasticity **for the Standardized Residuals**
